# Sexual patterns and practices among men who have sex with men and transgender women in Thailand: A qualitative assessment

**DOI:** 10.1371/journal.pone.0219169

**Published:** 2019-06-27

**Authors:** Pich Seekaew, Sita Lujintanon, Praditporn Pongtriang, Siriporn Nonnoi, Piranun Hongchookait, Sumitr Tongmuang, Yarinda Srisutat, Praphan Phanuphak, Nittaya Phanuphak

**Affiliations:** 1 PREVENTION, Thai Red Cross AIDS Research Centre, Bangkok, Thailand; 2 Faculty of Nursing, Suratthani Rajabhat University, Surat Thani, Thailand; University of Westminster, UNITED KINGDOM

## Abstract

The global trend in HIV incidence overall is declining; however, there is a plateau in new HIV infection among men who have sex with men (MSM) and transgender women (TGW) despite extensive investment in HIV prevention targeting these populations. Many studies usually conflate these two groups together, which may overlook many disparate characteristics unique to each population, impeding the efficacy of HIV interventions. To better understand the vulnerable diversity that may put these individuals at risk of HIV infection, we conducted qualitative analysis among Thai MSM and TGW, aiming to identify sexual pattern themes of MSM and TGW in Bangkok in order to better understand their distinctive sexual life context. Convenient and purposive samplings were used to recruit Thai MSM and TGW aged ≥ 18 years old and living in Bangkok, Thailand, for focused group discussions and one-on-one in-depth interviews, respectively. Total of 12 MSM and 13 TGW participated in focused group discussions, which were conducted separately for MSM and TGW. Additionally, 5 MSM and 5 TGW were involved in one-on-one in-depth interviews. Thematic analyses were performed separately for MSM and TGW. The results show that MSM and TGW have distinct and diverse sexual patterns, and within the identified themes: partnering, partner finding, protection, and enhancing sexual pleasure (only for MSM). Participants reported having varying sexual experiences. Recognizing the difference and diversity in partnering and sexual practice of MSM and TGW is crucial in order to develop tailored interventions that suit the vulnerability of the key populations in Thailand.

## Introduction

With the surge of globalization, Thai sexual norm, particularly among younger generations, is changing rapidly, resulting in a decline in the age of sexual debut, an increase in the number of sexual partners, and an increase in acceptance of premarital sexual intercourse [1]. This effect may be greater among men who have sex with men (MSM) than heterosexuals and may help to explain the high incidence of HIV among MSM, since they tend to have an earlier sexual debut and a higher number of sexual partners, throughout the lifetime and concurrently [2]. MSM and transgender women (TGW) face many barriers, such as the difficulty accessing health or social services, economic marginalization, and discrimination and exclusion from the mainstream gender binary society [3–5], making them more vulnerable to HIV infection. MSM and TGW are at the heightened risks of 19.3-time [6] and 48.8-time [4] respectively, when compared to the general population, and they disproportionately contribute to more than 50% of all new HIV infections in Thailand annually, even while the number of new HIV infections in Thailand has been decreasing [7]. Thus, this calls for more effort in developing targeted interventions for these populations in order for Thailand to end its HIV epidemic.

MSM and TGW are a diverse group of people, not only on the basis of gender identity and sexual orientation, but also on the basis of age, ethnicity, socioeconomic status, and their engagement in HIV-vulnerable activities [5]. These variables vary and present different HIV-risk levels [5]. Despite the diversity, stigma and discrimination are their common denominator due to homosexuality, which plays an important role in their lives and their decision in engaging in vulnerable behaviors [8, 9]. Where and how MSM and TGW interact with their sex partner may also vary, and their sex culture is constantly changing, which may become a challenge for those who are outside of their community to understand and work with them. Additionally, there is a limited amount of research conducted on TGW. Most research on MSM and TGW is conducted in the Western world, and sex culture is known to be varied with geographical contexts [10, 11].

Understanding the sexual life context of the most affected population here is imperative to end the HIV epidemic in Thailand. Thus, this manuscript aims to examine patterns that may be able to describe their sexual behavior, sexual preference, and other risk factors that contribute to HIV infection among Thai MSM and TGW.

## Materials and methods

### Participants

Participants who self-identified as MSM or TGW and were at least 18 years old were asked to participate in this study. Convenient sampling was used, through face-to-face and telephone, to recruit the participants, and the recruitment was conducted in Bangkok, Thailand, at Thai Red Cross Anonymous Clinic (TRC-AC), Rainbow Sky Association of Thailand (RSAT), and Service Workers in Group Foundation (SWING). TRC-AC is the largest facility-based HIV and STI testing center in Thailand, offering comprehensive sexual health services catering to all populations. SWING and RSAT are community-based, key population-led health service centers, offering HIV and STI testing, and sexual care specifically for MSM and TGW populations. Potential participants were informed about the study, and those who agreed to participate signed an informed consent form. Additionally, purposive sampling was used to recruit participants who participated in focused group interviews to partake in one-on-one in-depth interviews.

### Data collection and analysis

Demographic data (age, religion, sexual orientation, marital status, employment status, income level, and education level) were collected prior to the interviews. Semi-structured, topics-guided focused group discussions were conducted among 12 MSM and 13 TGW, separately, on February 9, 2017, and took place at TRC-AC. The domains outlines were: history and experience of sexual encounters, experience of relationships, and health literacy on and experience of HIV prevention. Each discussion session lasted approximately 60–90 minutes. Additionally, 5 MSM and 5 TGW from the current participant pool were asked to participate in additional one-on-one in-depth interviews. The purpose for additional in-depth interviews was to allow participants to discuss certain sensitive topics in further details that may be prohibitive in a group discussion setting. Probes emerged within each domain were used to guide in-depth interviews. One-on-one in-depth interviews took place between February 10, 2017, and February 20, 2017, and were conducted in a private meeting room at TRC-AC. All interviews were led by 2 PhD- and MPH-trained, Thai-native researchers. All interviews were audio-recorded, transcribed, and translated verbatim by a professional translator. All personal signifiers were removed to ensure anonymity. Some Thai and English transcripts were randomly selected and reviewed by our research team to ensure the accuracy of the data.

The data analysis was performed manually, and we followed the framework analysis outlined by Braun and Clarke [12]: After carefully reviewing the transcripts in their entirety, a code book was created and the transcripts were manually coded by 3 research team members independently. Once the transcripts were coded, the team members met and discussed the potential overarching themes of the transcripts, and eventually categorized into major themes and subthemes. Then, data extracts were selected to demonstrate the themes. To ensure the rigorous of the analysis, the transcripts and the themes were reviewed and commented by other research team members prior to finalizing the data. The thematic analysis was conducted separately for MSM and TGW participants.

For demographic data, statistical analysis was performed with Stata version 14 (Statacorp, College Station, TX). Characteristics of the participants were summarized using frequency, simple proportions, and medians (IQRs) based on the nature of each variable and stratified by population. To compare the differences in outcomes between population groups, Fisher’s Exact test was used for categorical variables, and Kruskal–Wallis test was performed for continuous variables.

### Ethical concern

This research project was approved by the Human Research Ethics committee at Chulalongkorn University (IRB:158/56; NCT:02437981). Each participant was provided the information statement and informed consent explaining the nature of the study, including methods, interview process, as well as possible benefits and risks. Those who decided to participate signed the consent form. Participants could refuse or withdraw to participate at any time.

## Results

### Participant characteristics

Of 25 participants, 12 were MSM and 13 were TGW. The median (IQR) age for MSM and TGW were 33.1 (29.9–35.7) and 25.8 (23.4–29.1), respectively. Among MSM participants, 41.7% (5/12) identified themselves as male, while 58.3% (7/12) identified as either lesbian, MSM, or bisexual. In contrast, 23.1% (3/13) of TGW participants identified themselves as female, and 69.2% (9/13) identified as transwoman. Of all MSM, 41.7% (5/12) lived with their regular sexual partner; 76.9% (10/13) of TGW were single. In addition, 75% (9/12) and 69.2% (9/13) of MSM and TGW had at least a college degree and above, respectively (Table 1).

**Table 1:** Demographics of MSM and TGW participants.

Characteristics	Overall (N = 25)	MSM (n = 12)	TGW (n = 13)	P-value
Age(years)				
Median (IQR)	29.6(23.6–34.1)	33.1(29.9–35.7)	25.8(23.4–29.1)	0.07
Religion				-
Buddhism	23(92)	11(91.7)	12(92.3)	
Christian	1(4)	0(0)	1(7.7)	
Islam	1(4)	1(8.3)	0(0)	
Gender Identity				-
Male	5(20)	5(41.7)	0(0)	
Female	3(12)	0(0)	3(23.1)	
Transwoman (male-to-female)	9(36)	0(0)	9(69.2)	
Others: Lesbian, MSM, Gay, Bisexual	8(32)	7(58.3)	1(7.7)	
Marital status				0.003
Living together with regular sexual partner	6(24)	5(41.7)	1(7.7)	
Have the regular sex partner but not living together	6(24)	4(33.3)	2(15.4)	
Single	11(44)	1(8.3)	10(76.9)	
Others	2(8)	2(16.7)	0(0)	
Employment status				0.88
Full-time	9(36)	4(33.3)	5(38.5)	
Part-time	8(32)	3(25)	5(38.5)	
Unemployed	7(28)	4(33.3)	3(23.1)	
Missing	1(4)	1(8.3)	0(0)	
Income (THB^1^/month)				
Median (IQR)	17,500(13,500–26,250	25,000(9,000–50,000)	15,000(15,000- 20,000)	0.10
Education				0.37
Primary school	1(4)	0(0)	1(7.7)	
Secondary school/High school	4(16)	1(8.3)	3(23.1)	
Diploma	2(8)	2(16.7)	0(0)	
College/University or above	18(72)	9(75)	9(69.2)	

^1^ THB = Thai Baht

### Sexual pattern of MSM

Analysis of the interview transcripts identified four themes related to sexual practices among MSM: (1) partnering, (2) partner finding, (3) protection, and (4) enhancing sexual pleasure (Table 2).

**Table 2:** Themes for MSM participants.

Theme	Subtheme	Quotes
Partnering		
	Open Relationship	MSM: Well, I tell [my regular sex partner] when I have sex with some [other men], and sex stories become something that we share and amuse ourselves. For example, [we would ask each other], I didn’t see you yesterday, where did you go? Who did you have sex with? Show me his body. Then we would exchange and tease each other about having many sex partners. It is the fun in life.
	No primary partner	MSM: I see sex as for fun. After we are done, if I want to have sex with you again, we will, but I don’t want to tie myself to you. I don’t know. MSM: I have a motto, after cumming, let’s just part ways, no more second time, that’s the kind of person I am. If I have sex with you, I will only have sex with you once. If you want to stay connected and be friends, I will not be in mood for… Interviewer: Normal [relationship]? MSM: Yes, I don’t want to see him. I feel like why do we have to date each other? Why do we have to get to know each other?
	Increased number of partners when closeted	When I was closeted, I wondered why I had so many more partners. In a year, I had almost 200 partners, some year, 160 partners. I have proof because I kept a diary about my sex life. My friend would tease me, who would be my next number? I would come back at him, saying that if you want to be my next number, let me know, tell me which month, which number you want to reserve.
	Group sex	I don’t know, I like [group sex], but before, I was shy. Later, it was nothing. Before, when I had group sex with my friends, I felt that I didn’t like having sex with someone I didn’t want. Do you understand? Because I didn’t know what they were going to look like. I would have to gamble [that I would met someone that I liked]. But now, if I pay for sex and I get to have a group sex, I consider it as a bonus.
Partner finding		
	Venues	Interviewer: Normally, where do you find your partner? MSM: From gyms, malls, restaurants. I don’t normally go out at night. MSM: I like going to sauna, to be frank. When I go to Hong Kong or Japan, I will go to sauna. I went to the two countries and I had to go to sauna, I don’t know why. Interviewer: Why do you choose to go to sauna? MSM: Because I feel that I don’t need to [put myself out there to seek for partners]. I view it as a private space and a place that I don’t need to say anything, and other people will still know that I am gay, because normally I am closeted. Interviewer: Where did you recruit people to have group sex? MSM: Hornet. Interviewer: Was it the only way? MSM: Hornet, Jack’d and Grindr. These three mobile applications.
	Gender preference	A transgender woman took a liking in me once but I told her, I don’t like transgender women. But I have to admit that I like how transgender women take care and are attentive, because I had a transgender woman doing that to me, and it felt good. But if asked if I was turned on, over my dead body, I never thought I would be. Interviewer: Are you attracted to women? MSM: If I was with her, I could flirt with her. But looking for sex, probably not. Interviewer: Have you had a girlfriend? MSM: I have. Interviewer: But you didn’t have sex with her? MSM: Yes. Before my current boyfriend, I was seeing both a guy and a girl at the same time. Interviewer: How did that feel? MSM: It was exciting. It was fun for me. I felt like I could switch whenever I wanted. For instance, if I was lonely, I would go see the girlfriend. I would return to the boyfriend when I wanted to.
	Sexual position preference	MSM: When I meet someone of my type, I have to say frankly, I am crazy about guys with light skin, Chinese look, and muscular and fit body, I can’t say no to these guys if I meet one. I will give in, but I won’t be on the receptive side. Interviewer: Do you top only? MSM: I always know that I will conquer other guys but I will never be conquered. I have my standpoint that I will never bottom. Even when I went to Hong Kong, even when I found guys with light skin [like my type], I still wouldn’t allow. Interviewer: How do you decide whether you will top or bottom? The person that you will top and the person that will top you, are they different? MSM: I don’t know. Normally, when I meet someone, they always let me decide. Sometimes if he never bottomed, he would bottom for me. Sometimes he never topped, he would top me.
	Sex in exchange for valuables	When I meet someone older than me, I will tell him, you have to “pay” for me. If you “pay” for me, I’m okay. I put it straight. I’m clear, for example, during the new year, he sent me a plane ticket to Krabi, but once I got there, he didn’t take a liking in me, so I took the flight back. I wasn’t serious about it. Interviewer: Have you loved a man? MSM: I have, but he didn’t love me. It was full of disappointment. So I [think], no more [love]. I’m this old, if I want sex, I will pay for it.
Protection		
	Decision to use protection	Interviewer: No matter what, you have to use condom with your boyfriend? MSM: Yes, so he would not feel guilty if I contracted HIV from him. Because he told me that, if that happened, he would never forgive himself. Interviewer: So the one that you said you didn’t use condom with was your regular partner? MSM: Yes, regular partner. Interviewer: Besides this regular partner, do you have other partners that you think may be a potential risk? MSM: I don’t think so because besides my regular partner, with other partners, I always use condom.
	Consistency in using protection	Interviewer: Has someone asked you like, hey, let’s do it bareback? MSM: That has happened, but I refused or I just walked out. [There was one time] I was already in the room, and he said, you should take the condom off, [and I said], no and I walked out. I always feel bad if I don’t use condom. However, on that day, [the condom] slipped off, it really was an accident. Interviewer: Why did you have bareback sex with him? MSM: I was the one doing it. Interviewer: In that occasion, why? MSM: I had some beer and I didn’t notice that I finished inside. I thought he was just another casual sex partner, and I might finish in him. Interviewer: Was he shocked? MSM: He wasn’t. He said he wanted to bareback. Interviewer: Did he tell why barebacking is better? MSM: He said, we are boyfriends. It was not just lust. It was a romantic fantasy like in The Fifty Shades of Grey, two people loved each other and had raw and passionate sex. My behavior, do I think it is risky? I accept that my behavior is risky but I have a moral sense that no matter what I have to wear condom. But if I don’t wear condom, I can say it is oral sex. I am a million percent confident to say that for oral sex, I have never worn a condom.
Enhancing sexual experience		
	Drug use	Interviewer: How did it feel when you smoke weed? MSM: When we were having sex, it felt like we were in the space. Everything was a fantasy. Even the semen that was ejaculated, I saw it as something beautiful. It wasn’t gross or anything. It was a fantasy. I was happy. I can’t really describe it. Popper is something that when I have sex, I’m not sure if I’m addicted to it or not, but without popper, I don’t feel the sexual desire.
	Wanting to test positive	MSM: I want to test [HIV] positive because, firstly, I love him and I don’t want him to feel alone. Another thing so that sex will be more convenient. Interviewer: Does convenience mean fun? MSM: More fun.

#### Partnering

The level of commitment to the regular partner and the acceptability of concurrency among MSM are lower and higher, respectively, when compared to heterosexual relationship. These allow multiple forms of partnering ranging from an open relationship with one committed regular partner to a short-term relationship without emotional tie, including one-night stands. In the theme of partnering, 4 subthemes emerge: open relationship, no primary partner, increased number of partners when closeted, and group sex. While some MSM had a regular partner, they mutually agreed to have an open relationship, meaning that each partner could have sexual partners outside their relationship. One MSM participant even shared that he and his partner enjoyed sharing anecdotes about their sexual encounters with each other, so they were aware of the sexual activities going on outside of their relationship. Some MSM did not have a primary partner, meaning that they were not in a committed relationship but have sexual activities with casual partners, including having a series of one-night stands. Some MSM participants approached this subtheme with a positive attitude that, to them, sex represents fun, and without a primary partner, they had the freedom to have fun. However, some MSM participants could not stand having a committed or emotionally involved relationship or even form a friendship with their one-time sexual partner, so they chose to have no primary partner. Nonetheless, having an open relationship and having no primary partner result in a high number of sexual partners among MSM. Interestingly, they also noted that they found more partners when they did not disclose their gender identity. Group sex was also quite a common practice among MSM participants. It could be either an open group, which anyone could join, or a closed group in which all participants knew each other.

#### Partner finding

Partner finding depends on gender preference, sexual position preference, preference for sex in exchange for valuables, and the venues the MSM frequent. While most MSM were only involved with other MSM, a few MSM participants were interested in women and TGW; however, they only had platonic relationships with them. Still, this demonstrates the diversity in sexual orientation among MSM. Sexual position preference helps narrow down compatible partners. MSM in the study reported preferring only being insertive or versatile when having anal intercourse; none mentioned that they preferred being receptive only. Some participants were lenient about their preferred position, while others were very adamant. In addition, some MSM participants revealed that they liked oral sex more than anal intercourse. The venues of partner finding are important because they did not only determine where MSM would meet but also the type of partners they would find. MSM participants used both offline and online venues to find partner; however, from our finding, offline venues were limited to finding casual partners and could be divided into day- and night-time venues. Day-time venues included gyms, malls, and restaurants; night-time venues included traditional venues to find sex partners such as nightclubs and bars as well as men-only massage parlors and saunas. In addition, massage parlors were common venues for sex in exchange for money and/or valuables in which our MSM participants were found on both buyer and seller sides. MSM participants that have bought sex before revealed that they paid cash for sex, and different sexual services had different rates. For the sellers, they did not necessarily ask for cash but they often asked the buyers to pay something for them instead. Lastly, MSM took the advantage of the rising era of internet and online partner selection applications to find both casual and permanent partners, and even partners for group sex.

#### Protection

Our MSM participants were highly aware of the use of protection, including the use of condom and lubricants. However, the actual use of protection might vary depending on circumstances. For some, the decision to use condom depended on partner type, either regular partner or casual partner. Some MSM used condom when having sex with regular partner and reasoned that they would feel guilty if they transmitted HIV/STI to their partner, so they needed to take precaution and protect their partner. On the other hand, some MSM would not use protection with their regular partner, but would use with casual partners. This was their strategy to prevent HIV/STI transmission from casual partners and increase intimacy between regular partners. Additionally, many MSM who participated in group sex recognized the risk of having sex with multiple partners or strangers and tended to take precautions, such as setting a rule that everyone must wear condom and being on PrEP. Among regular condom users, the consistency of using condom was quite high. Some MSM would even refuse to have intercourse without condom. Nonetheless, there were exceptions, such as accidental slips, sex under influence, and oral sex.

#### Enhancing sexual pleasure

Many MSM turned to alcohol and drugs in order to enhance their sexual drive, sexual performance, and sexual experience. The drugs mentioned in the interviews include cannabis, popper, amphetamine, and cocaine. Some MSM have become reliant on these drugs and lost their sexual drive without them. Moreover, to enhance the sexual pleasure, some MSM in serodiscordant relationship had an idea of wanting to become HIV positive in order to empathize with their partner and to be able to have a spontaneous sexual encounter without worrying about protection or HIV infection, though this has yet to be seen in action.

### Sexual pattern of TGW

Analysis of TGW transcripts identified similar themes: (1) partnering, (2) partner finding, and (3) protection (Table 3).

**Table 3:** Themes for TGW participants.

Theme	Subtheme	Quotes
Partnering		
	Concurrency	Interviewer: When you had sex with him, who started? TGW: Him. He wanted because he was stressed out from work, and I gave in Interviewer: Did your boyfriend know? TGW: My boyfriend didn’t know. Interviewer: Did you use condom? TGW: I always use condom Interviewer: Do you think your boyfriend has had sex with other people? TGW: I think, there was one time he went out, I think he had someone, sometimes, but he has never made me worry, so I didn’t say anything Interviewer: Do you accept this? TGW: I’m okay. He didn’t love that person, he loves me. Since he lives with me, he takes a good care of me, so I’m okay.
	No primary partner	I don’t want to be in the world of relationship full of nonsense, full of jealousy, anymore. I’m done. Let’s have sex and that’s it. If we see each other again in a while, we can have sex again.
Partner finding		
	Venues	TGW: BeeTalk is like a mobile application that you can chat with people, like for example, if I found that you were online, I could add you as a friend and we could chat, something like that. Interviewer: Is this app also a way to find sex partner? TGW: Part of it. I found many Interviewer: So you do use it [to find sex partner] TGW: Yes
	Sex in exchange for valuables	Interviewer: When you were in a relationship [with your boyfriend], did you have sex with other people? TGW: I had a sugar daddy. But there wasn’t much, I went to see him, got food with him. I had sex with him but I used condom.
Protection		
	Decision to use protection	TGW: I always use condom, I always use condom with my boyfriend. Interviewer: Has you boyfriend asked for condomless sex? TGW: He hasn’t. I asked him why he has to have protected sex with me, and he said that he doesn’t trust himself and he had many [risky] sexual encounters in the past. Interviewer: Does your [casual] partner use condom? TGW: Yes Interviewer: What if he didn’t use condom? TGW: I wouldn’t have sex with him. Interviewer: You wouldn’t have sex with him TGW: I use condom with him because he is not my boyfriend. Interviewer: Are you worried when you have sex with someone? TGW: I’ve been worried since I moved to Bangkok Interviewer: What are you scared of? TGW: AIDS. I’m in Bangkok so I’m scared, I wasn’t that scared when I was back in Korat. Interviewer: So when you met someone via the mobile application, you didn’t use condom? TGW: I used condom. When I’m in Bangkok, I always use condom. Interviewer: Who told you about condom? TGW: When we are in Bangkok, we have to use it. The [casual] partner, he also uses it.
	Consistency in using protection	Interviewer: For the time you didn’t use condom, why didn’t you use it? TGW: I was in a hurry. Interviewer: Why were you in a hurry? TGW: There wasn’t enough time and there was no condom. There was nothing. But he pulled out and ejaculated outside. It was safer than cumming inside. TGW: I didn’t use condom. Interviewer: Why? TGW: I was young and I wasn’t taught about safe sex
	Condom breakage	Interviewer: What’s the riskiest thing you have done? TGW: Condom breaking Interviewer: You used lube and wasn’t doing anything rough, right? TGW: Yes, I think it was friction, because we didn’t cover condom with lube, that caused the condom to break

#### Partnering

Two subthemes emerged for TGW partnering: concurrency and no primary partner. Similar to MSM, concurrency, meaning having more than one sexual partner at the same time,was high among TGW in a relationship. However, there was no agreement about the nature of the relationship between TGW and their regular partner. We found that most TGW participants did not inform their regular partner about casual partners, even though they had a close relationship with their regular partner that they spoke fondly of him and addressed him as a boyfriend. TGW participants also did not confront their regular partner about his casual partners. Nevertheless, some TGW chose not to be in a relationship and had no primary partner because they wanted to avoid relationship issues. Thus, they opted to have casual sex instead.

#### Partner finding

The pattern of partner finding among TGW is quite uniform in comparison to that of MSM. Their gender and sexual position preferences were homogeneous, as they reported to only have sex with men and engage mainly in receptive anal sex and oral sex only. Partner finding through online platform was the most preferred method among TGW for both regular and casual partners. BeeTalk was among the popular partner-seeking mobile applications that TGW used to connect with potential partners. For sex in exchange for valuables, TGW were only found on the side of receiving valuables for sex. Similar to MSM, TGW were able to negotiate their deal and sometimes did not ask for cash; instead, they requested the “customers” to purchase material things and to pay their bills.

#### Protection

In comparison to MSM, there were more TGW who had poor knowledge about safe sex and protection. Some TGW participants who were not Bangkok natives mentioned that they were not taught about condom when they were young, or they were aware of condom but did not use it until they moved to Bangkok where they met partners that actively used protection. This led to poor decision making in using protection. Similar to MSM, the decision to use condom depended on the type of partners. Some TGW chose to use condom with their regular partner, while others chose to use condom with casual partners but not with regular partner. But for commercial sex, TGW always used condoms with clients. From our cohort, many TGW were worried and fear about contracting HIV, and this prompted them to use condom more regularly. Nonetheless, inconsistency in using protection still occured. Failure to use protection was reported more often among TGW and could be attributed to limited knowledge pertaining HIV prevention, poor judgement during sexual impulsivity and arousability, and false trust in partner sexual history. In addition, some attempts to use protection failed because of inadequate lubrication, leading to the problem of condom breakage.

## Discussion

Our study identifies sexual pattern themes from interview transcripts of MSM and TGW in Bangkok; however, MSM and TGW were found to have diverse experiences with partnership and sex within the identified themes: partnering, partner finding, protection, and enhancing sexual pleasure (only for MSM).

Having multiple partners is quite common for MSM and TGW. Similar to our finding, MSM in Hanoi, Vietnam, reported a high number of partners up to the hundreds [13], and having multiple sexual partners may help maximize sexual pleasure [14]. Encounters with these partners may be serial or concurrent [2]. A stable, committed, monogamous relationship with intimacy and trust between the two partners in the traditional sense of heterosexual couples is rare [13, 15] and is not reported in this study. Though MSM and TGW do form a committed relationship with one partner, their relationship is often open, meaning that they may have partners outside of the relationship [15, 16]. In addition, there are varying degrees of openness in the relationship as agreed between partners, with MSM seem to be more transparent with their partner than TGW. This may suggest that MSM have equal power in the relationship, while TGW may be more passive and dependent on their boyfriend, taking a stereotypical feminine role in a relationship [17].

Additionally, MSM participants revealed a diverse preference for partner gender, sexual position, meeting venues, and sex in exchange for valuables, whereas the preference of TGW participants was quite uniform. While all TGW participants had sex and relationship with only men, some MSM mentioned having relationship with women and TGW. Having a masculine external appearance may allow MSM to live in the masculine heterosexual society [17], allowing them to experiment more with their gender identity and sexual attraction. Our findings also reveal that gender identity and sexual orientation determine where MSM and TGW could interact with potential partners. MSM outward appearance and the availability of MSM tailored venues, such as gay bar and sauna, permit MSM to find partners in the offline platform. TGW, on the other hand, have limited offline opportunity. Nonetheless, both populations take the advantage of the online partner-seeking applications, which become the main outlet for MSM and TGW to find partners. Online venues make partner finding more convenient [14] for sexual minorities and marginalized people to openly connect with potential partners [18]. In fact, MSM and TGW in our study used online venues to find all types of partners: serious, casual, or partner(s) for group sex. Though there is no pattern found in which online partner finding led to partaking in higher HIV-risk behaviors, other studies did find that there are downsides to online partner finding: online partner finding may lead to more multiple partnering and meaningless, casual sex [14, 19]. Previous studies also reported that MSM who use online partner selection applications are more likely to have condomless sex, use drugs, and test positive for HIV when compared to MSM using offline venues [19, 20]. Nonetheless, both offline and online venues identified as “pre-dating zones” serve as potential places for public health intervention, and it is particularly imperative to have interventions that target those who use the novel online venues. Additionally, it is also possible to have interventions be beyond the realm of HIV prevention campaigns: a recent study conducted in Thailand found that online channels have high potential to support HIV testing, and may be able to support Post-Exposure Prophylaxis (PEP) and Pre-Exposure Prophylaxis (PrEP) delivery in the near future [21].

Despite ubiquitous risk, MSM have better knowledge of safe sex and practice proper use of condom more often than TGW, especially TGW who are not Bangkok natives, though they have become more active in using protection since relocating to Bangkok. This shows that people in Bangkok in general are more health conscious and more active in using condom, and the decision to use condom of individuals in a community can influence others’ decision. Therefore, increasing HIV prevention awareness and practice for all men who have sex with TGW as well as TGW themselves is crucial in order to expand condom use among TGW community, especially since our finding reveals that the decision to use protection depends on partner type or the partner. Due to the difficulty in finding an understanding partner, TGW desire to be in a relationship with a regular partner may make TGW forgo condom use and willing to sexually engage with their partner, who may request HIV-vulnerable behaviors [8, 17]. This false sense of safety and protection ultimately put TGW at an increased risk of contracting HIV and STI. A holistic intervention to empower TGW is necessary, and TGW-specific interventions are urgently needed to increase HIV care uptake; for instance, offering integrated and gender-affirming transgender care and services [22].

Our analysis indicates that engaging in concurrent relationships is common for both populations, with inconsistent condom use patterns depending on the partner type, which includes partners for group sex for MSM. Interestingly, while group sex may be perceived as a potential HIV-risk behavior, our MSM participants seemed to be more proactive about using protection when having group sex. One study found a similar result that men who have both group sex and dyadic sex with men tend to use condoms during group sex more than dyadic sex. Researchers reasoned that group sex provided less opportunities for MSM to discuss issues concerning HIV [23], consequently, causing MSM to take extra precautions to prevent HIV transmission. Moreover, a few MSM in our study expressed their desire to become HIV-positive in order to empathize with their HIV-positive partner and to enhance sexual pleasure through condomless sex. This is a problem that is not found in other literatures. Though this has yet to become an action, it suggests the insufficient understanding of HIV and the burden associated with having the virus. Though recent studies found that persons living with HIV with undetectable viral load (VL) cannot transmit the virus [24, 25], the health literacy on this concept is limited, and viral load status is neither easily known nor commonly discussed among Thai communities. In addition, VL may not be routinely monitored or monitored much less frequently in resource-limited settings when compared to resource-rich countries [26, 27]. Furthermore, ART coverage even among those with known HIV-positive status is still low in these settings [28, 29]. Thus, we cannot solely rely on this principle. Hence, PrEP should be promoted more widely to let these misinterpreted MSM who wanted to test positive know that there is an alternative and, more importantly, PrEP should be the key intervention tool for Thai MSM as well as TGW until we achieve better coverage for the 2^nd^ and 3^rd^ 90 targets.

There are several limitations in our study. The subject of the interview is sensitive and participants might try expressing their view and sharing their experience in a socially acceptable way. Thus, social desirability may be present in our findings. Since our analysis is based on interview scripts of 25 MSM and TGW participants, generalizability is limited, though we reached the point of data saturation during the analysis. Follow-up studies with a large sample size and a quantitative analysis are required to confirm sexual patterns found in this study. Sexual culture is also constantly changing and varied with geographical context, so our findings may only describe sexual culture of MSM and TGW in Bangkok in this time. Nonetheless, our study identifies sexual pattern themes that put their HIV risk behaviors in real life context and enhance the understanding of MSM and TGW sexual cultures. This ultimately will help locate future interventions in order to work toward ending Thailand’s HIV epidemic.

## Conclusions

MSM and TGW have long been lumped into the same category, most often labeled as male-to-male sex. This overgeneralization overlooks the uniqueness that underlies the vulnerability encompassing these individuals. In each major theme, we found great differences between the two populations, granting us an insight into why many of our current tunnel vision interventions are barely effective. Past literature has identified TGW are at much greater risk to acquire HIV when compared to other populations, including MSM. This study found unequal power dynamic against TGW, and inadequate health literacy and safe sexual practice are great barriers to reduce HIV transmission among this population. Additionally, Thai MSM encounter issues concerning drug use and group sex similar to their counterparts in the Northern hemisphere, yet few studies are being conducted in the Thai context.

Future studies that are gender-specific must be done to mitigate the disproportionate burden these key populations are facing, including using online channel as an entry point to sexual health cascade and a platform to deliver interventions, as it can reach the otherwise uncaptured individuals.

## Supporting information

S1 File Topics for semi-structured interview (English). (DOCX)

S2 File Topics for semi-structured interview (Thai). (DOCX)
