# Supplementary material for: Sexual patterns and practices among men who have sex with men and transgender women in Thailand: A qualitative assessment
Source: PLoS One. 2019 Jun 27;14(6):e0219169. doi: 10.1371/journal.pone.0219169 (PMC6597192; doi:10.1371/journal.pone.0219169)
Supplement: S1 File — (DOCX) [file pone.0219169.s001.docx]

**Topics for semi-structured interview are categorized into 3 main topics to be used for group discussions and in-depth interviews.**

| 1 | **History and Experience of Sexual Encounters** |
| --- | --- |
| 1.1 | Thoughts and opinion towards sex |
| 1.2 | Sexual attraction and sexual behavior |
| 1.3 | First sexual encounter |
| 1.4 | Methods for seeking sexual partners |
| 1.5 | Considerations for and factors associated with seeking sexual partners |
| 2 | **Experience of Relationships** |
| 2.1 | Definition of being in a relationship |
| 2.2 | Experience of being in a relationship |
| 2.3 | Methods for seeking partner(s) for a relationship |
| 3 | **Health Literacy on and Experience of HIV Prevention** |
| 3.1 | Knowledge and understanding of HIV prevention |
| 3.2 | Experience and preference of HIV prevention methods |
| 3.3 | Challenges of using HIV prevention tool(s) |
