# Supplementary material for: Sexual patterns and practices among men who have sex with men and transgender women in Thailand: A qualitative assessment
Source: PLoS One. 2019 Jun 27;14(6):e0219169. doi: 10.1371/journal.pone.0219169 (PMC6597192; doi:10.1371/journal.pone.0219169)
Supplement: S2 File — (DOCX) [file pone.0219169.s002.docx]

**แนวคำถามแบบกึ่งโครงสร้าง (semi-structured interview) จำนวน 3 ข้อใหญ่ เพื่อใช้ในการสัมภาษณ์เชิงลึกรายบุคคล และแบบกลุ่ม**

| 1 | **แนวคำถามเกี่ยวกับประวัติและประสบการณ์การมีเพศสัมพันธ์** |
| --- | --- |
| 1.1 | โปรดเล่าถึงความคิดหรือทัศนคติต่อการมีเพศสัมพันธ์ |
| 1.2 | โปรดเล่าถึงความชอบทางเพศ หรือ แรงดึงดูดทางเพศ ของคุณ |
| 1.3 | โปรดเล่าถึงประสบการณ์ครั้งแรกกับการมีเพศสัมพันธ์ |
| 1.4 | คุณมีวิธีการหาคู่อย่างไรบ้าง |
| 1.5 | คุณคิดว่าปัจจัยที่ส่งผลต่อการคู่มีอะไรบ้าง |
| 2 | **แนวคำถามความคิดเห็นเกี่ยวกับความสัมพันธ์** |
| 2.1 | โปรดนิยามคำว่า ความสัมพันธ์ ตามความเข้าใจของคุณ |
| 2.2 | โปรดเล่าถึงความสัมพันธ์ ไม่ว่ากับแฟน หรือ คู่นอนของคุณ |
| 2.3 | คุณมีวิธีการหาคู่สำหรับความสัมพันธ์อย่างไรบ้าง |
| 3 | **แนวคำถามเกี่ยวกับความรู้ด้าน HIV** |
| 3.1 | โปรดเล่าถึงความเข้าใจเกี่ยวกับการป้องกันเวลามีเพศสัมพันธ์ของคุณ |
| 3.2 | โปรดเล่าถึงประสบการณ์และข้อท้าทายของการป้องกันเวลามีเพศสัมพันธ์ของคุณ |
| 3.3 | โปรดเล่าถึงข้อท้าทายของการป้องกันเวลามีเพศสัมพันธ์ของคุณ |
